# Organic devices based on nickel nanowires transparent electrode

**DOI:** 10.1038/srep19813

**Published:** 2016-01-25

**Authors:** Jeongmo Kim, Wilson Jose da Silva, Abd. Rashid bin Mohd Yusoff, Jin Jang

**Affiliations:** 1 Department of Information, Display and Advanced Display Research Center, Kyung Hee University, Dongdaemun-ku, Seoul 130-171, Republic of Korea; 2 Universidade Tecnologica Federal do Parana, GPGEI – Av. Sete de Setembro, 3165 – CEP 80230-901, Curitiba, Parana, Brasil

## Abstract

Herein, we demonstrate a facile approach to synthesize long nickel nanowires and discuss its suitability to replace our commonly used transparent electrode, indium-tin-oxide (ITO), by a hydrazine hydrate reduction method where nickel ions are reduced to nickel atoms in an alkaline solution. The highly purified nickel nanowires show high transparency within the visible region, although the sheet resistance is slightly larger compared to that of our frequently used transparent electrode, ITO. A comparison study on organic light emitting diodes and organic solar cells, using commercially available ITO, silver nanowires, and nickel nanowires, are also discussed.

In the last few years, the race towards a potential replacement for our commonly used transparent conducting electrode (TCE) has seen a dramatic increase in interest in which a lot of research groups, as well as the variety of materials and approaches, have been put forward. This is due to the increased demand of photonics and optoelectronics devices. Thus, it resulted in new n-type, p-type, and novel composite TCE materials, as well as an improved influx in the number of theoretical and modeling studies for a deep understanding of the new TCEs. Amongst the proposed TCEs, one must take into account numerous properties that are relatively important namely optical reflection, transmission, and absorption. Based on these distinctive features, several excellent materials have dominated the TCE industry, including fluorine-doped tin oxide (SnO_2_:F)^1^,^2^ and metal oxide/Ag/metal-oxide stacks, such as AZO/Ag/AZO^3^,^4^, pyrolytic tin oxide^5^, indium tin oxide (ITO)^6^,^7^, and indium zinc oxide (IZO)^8^,^9^. Among the aforesaid TCEs, ITO is considered the most popular since it has been employed in various optoelectronics devices such as solar cell^6^,^7^, diode^10^,^11^, transistor^12^,^13^, and sensor^14^,^15^. Nevertheless, one of the main shortcomings of ITO is that the price of indium is rapidly increasing.

A recent report from Technavio^16^ illustrates that the value of indium increased by 70% in the past few years. Hence, there is a strong push toward the development of a new type of TCE which is more cost effective, low temperature, environmentally stable, non-toxic, and processable over large areas. Several materials have attracted a great deal of attention with some promising results, such as carbon nanotubes^17^, graphene^18^,^19^, conducting polymer, as well as noble metal nanowires^20^,^21^,^22^,^23^. In the latter case, their synthesizing method can be realized by various approaches in order to form and grow the noble-metal nanowires^24^,^25^,^26^,^27^,^28^,^29^. Current synthesizing methods for nanowires are based on either being template-assisted, the assembly of nanoparticles or surfactant-mediated growth. Nonetheless, these techniques typically resulted in detrimental surface topography, low yields, large diameters, or low aspect ratios. Previous reports have demonstrated the preparation of ultrathin Au nanowires utilizing oleylamine-AuCl and oleylamine-HAuCl_4_ complexes in which the oleylamine served as solvent, reducing agent, and surface capping agent^30^,^31^. Even so, it is interesting to note that there remains a great challenge to directly synthesize ultrathin metal nanowires without the use of the above-mentioned methods.

To underline the significance of the present work, it is essential to have an overview of the past achievements in the development of transparent conducting electrode (TCE). Figure 1 collects the sheet resistance evolution on film thickness for various types of TCEs. Graphene films demonstrate the lowest resistivities (~5 × 10^−6^ Ω cm), followed by TCO/Ag/TCO films and Ag nanogrids (~5 × 10^−5^ Ω cm). The ITO is slightly higher at ρ_ITO_ ≈ 2–5 × 10^−4^ Ω cm, which is the lower range attainable by higher film thickness. The best ITO film today reach ρ_ITO_ ≈ 10^−4^ Ω cm for the thickness above 100 nm, which would shift the ITO curve slightly to the left. PEDOT:PSS film and Ag NWs demonstrate the highest resistivities. Thus, this deficiency will be addressed in the present work, and will be discussed in organic light emitting diode (OLED) and organic solar cells (OSC).

Hence, we report a facile synthesis of Ni nanowires (NWs) without the use of any surfactant, organic, or inorganic template (see Experimental Details). This proposed method is facile, highly reproducible, and easy to scale up. To the best of our knowledge, there has been no report of the direct preparation of such Ni NWs assemblies in solution. The as-prepared NWs exhibits excellent transparency with good crystalline and surprisingly comparable OLEDs performance compared with our commercially available ITO.

## Results and Discussion

**Figure 2:**
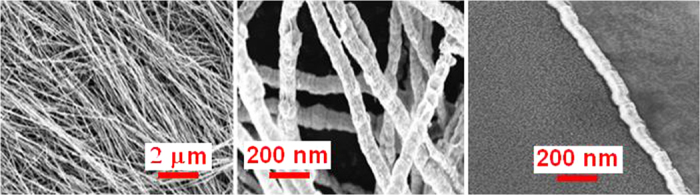
SEM images of purified Ni NWs with different magnifications.

**Figure 3:**
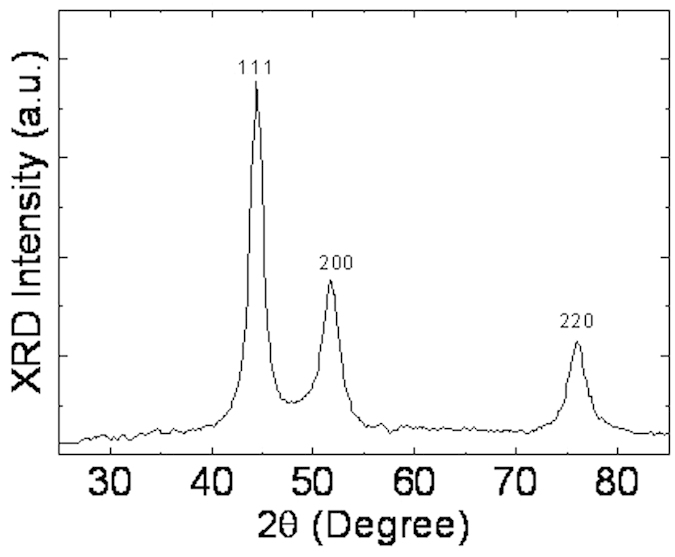
The XRD patterns of Ni NWs.

Figure 2 shows the morphology of the Ni NWs by scanning electron microscopy (SEM) with different magnification in which the diameters of Ni NWs ranged from 80 to 100 nm. Our Ni NWs were very long, with lengths up to approximately ~60 μm. Figure S1 illustrates the distributions of Ni NW diameters and lengths. Observably, the presence of short Ni NWs is from deteriorated long Ni NWs during the purification process. The XRD pattern of Ni NWs is shown in Fig. 3. There are three peaks of Ni that correspond to different crystallization directions of (111), (200), and (220) located at 44.4°, 51.7°, and 76° which indicated that Ni NWs has a cubic crystal structure.

**Figure 4:**
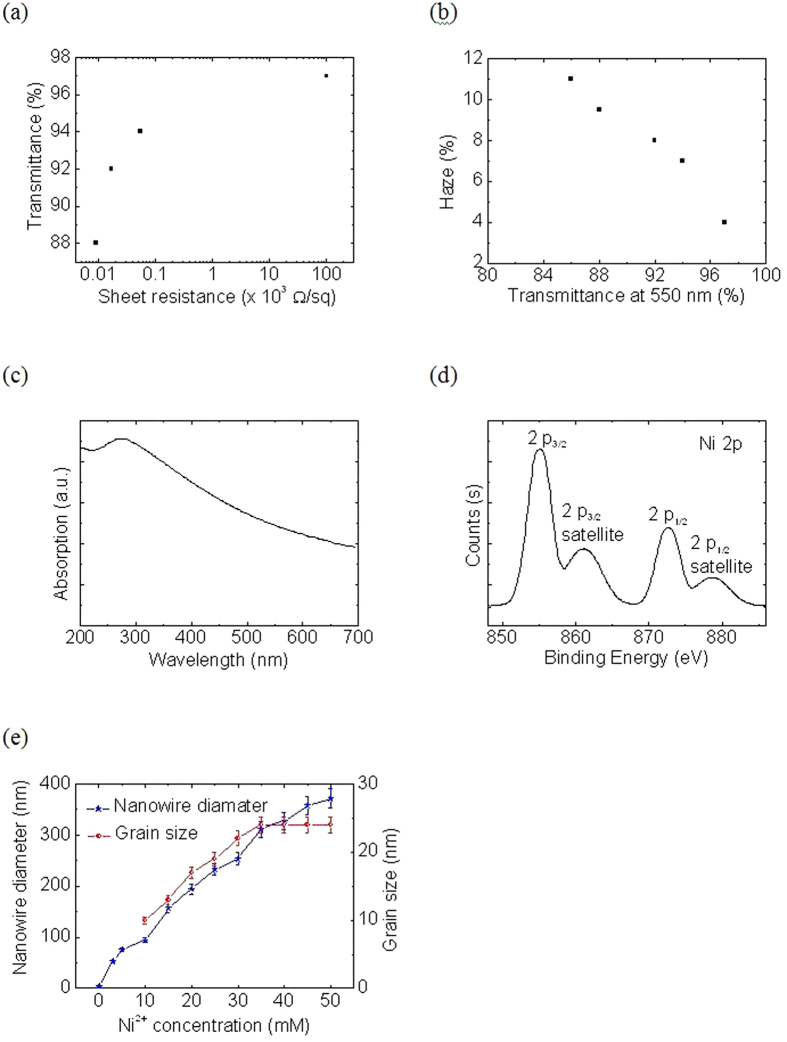
(**a**) Transmittance (at 550 nm) *vs* sheet resistance for Ni NW electrodes, (**b**) haze value *vs* transmittance of Ni NW electrode (at 550 nm), (**c**) UV-Vis absorption spectra of the Ni NWs thin film, (**d**) XPS core level of Ni 2p, and (**e**) the average diameter of Ni NWs and average grain diameter of Ni NWs *versus* concentration of Ni^2+^.

Figure 4 collects the important features of Ni NWs deposit of glass substrates. Figure 4a shows the transmittance at 550 nm *vs* the sheet resistance (R_S_). We obtained a good relation between the conductivity and transparency, specifically for the transparency below 90%. For the transparencies of 80%, 85%, 90% and 95%, our prepared Ni NWs demonstrate a R_S_ of 12, 14, 19, and 48 Ω/sq., respectively. We suspect that the percolation threshold of Ni NWs was exceeded which makes the electrodes less conducive for a transparency over 95%. Figure S2 illustrates the transparency *vs* Ni NWs, where the 90% transparency is roughly 0.035 Ni NW g.m^−2^. It is also worth noting that our approach does not require a high annealing temperature, which gives excellent compatibility with solution process devices, which are relatively sensitive to temperature. It is worth noting that the best quality of Ag NWs deposited on glass substrate had the lowest R_S_ of about 10.2 Ω/sq. along with 89.9% transparency^37^.

Meanwhile, Fig. 4b depicts the haze factor at 500 nm transmittance. Haze factor is a relatively important property for various optoelectronic devices^38^,^39^,^40^. Haze factor depends on Ni NWs network density in which an 11% haze value corresponds to approximately 85% transparency, whereas only 4% were obtained at a 97% transparency. This trend was expected since the high density of Ni NWs caused more light scattering. One should also note that there is no optimum haze factor value since it varies with applications. Figure 4c illustrates an absorption spectrum of Ni NWs thin film.

**Figure 5:**
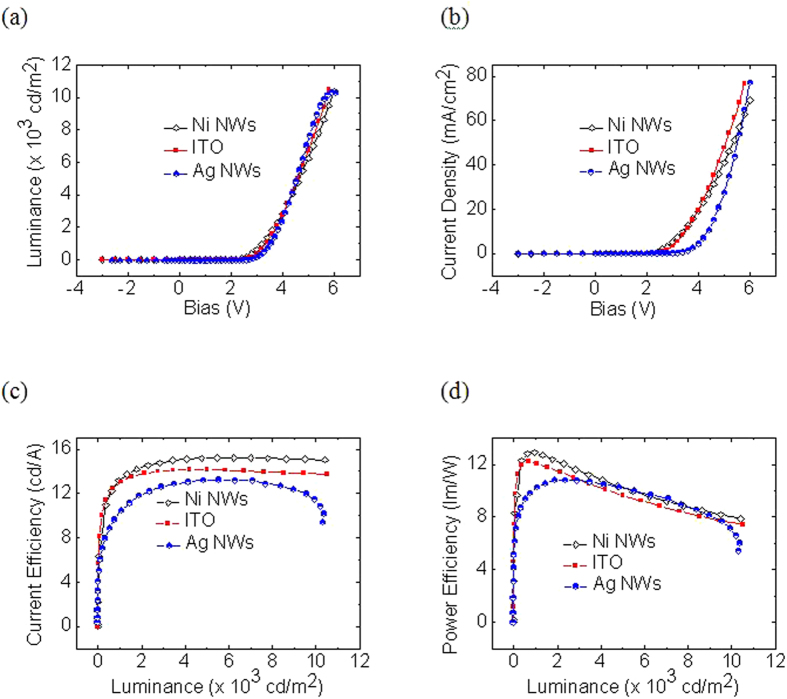
The (a) luminance-voltage, (b) current-voltage, (c) current efficiency-luminance, and (d) power efficiency-luminance characteristics of OLEDs fabricated based on ITO, Ag NWs, and Ni NWs as the transparent electrode.

In order to determine the chemical state of Ni NWs, X-Ray photoelectron spectroscopy (XPS) measurements were conducted (Fig. 4d). The binding energy of the most intense Ni 2p_3/2_ component of the Ni 2p doublet located at 855.5 eV corresponds to nickel oxide (Ni^2+^). In agreement with this is the observation of a satellite line, at about 8 eV higher binding energy of each Ni 2p component, which is the fingerprint of Ni^2+^ ions. It is likely that Ni NWs exposed to ambient atmosphere quickly become oxidized. Figure 4e shows the average diameter and average grain diameter as a function of Ni^2+^ concentrations. As one sees, the average diameter increases with concentration. It is worth noting that the Ni NWs prepared in this work are relatively smooth and uniform. The uniformity of NWs changed completely when the concentration was doubled (from 15 to 30 mM) and detailed regarding uniformity changes will be published elsewhere. On the other hand, the average grain diameter increases linearly and becomes saturated above 35 mM. We conclude that the prepared Ni NWs is efficient and provides an easy path to achieve high performance Ni NWs based TCEs. As we already have an excellent TCE, the next possible step is to implement the prepared transparent Ni NWs electrodes in OLEDs, as shown in Fig. 5.

Please note that besides fabricating organic devices based on Ni NWs and ITO based TCEs, we also prepared organic devices employing Ag NWs as the TCE. The basic architecture of the OLED consists of seven layers: Glass/Ni NWs/PEDOT:PSS (30 nm)/TAPC (20-nm)/10 ± 1 wt% Ir(2-phq)_3_:MTXSFCz (35-nm)/TmPyPB (55-nm)/LiF(0.9-nm)/Al (100-nm), where poly(3,4-ethylenedioxythiophene) (PEDOT) was used as the hole-injection layer (HIL), 1,1-bis[4-[N,N’-di(p-tolyl)amino]phenyl] cyclohexane (TAPC) was used as the hole-transporting layer (HTL), and 1,3,5-tri(*m*-pyrid-3-yl-phenyl) benzene (TmPyPB) was used as the electron-transporting layer (ETL) and hole-blocking layer (HBL). Figure 5a,b show the current density−voltage−luminance characteristics of the OLEDs prepared, using Ni NWs as the bottom electrode. The results were compared with the properties of a reference OLED, prepared with our commercially available Ag NWs and ITO. The OLEDs with an ITO electrode demonstrated a slightly higher operating voltage (Fig. 5c,d), marginally lower current density, and a lower luminous efficiency (14.16 cd/A and 12.20 lm/W) than the device prepared with Ni NWs (15.23 cd/A and 12.91 lm/W). The large electron injection energy barrier, from the ITO (with a work function of ~4.8 eV) to the PEDOT:PSS (with a lowest unoccupied molecular orbital, LUMO, energy level of ~2.2 eV), impeded electron injection and reduced the current density. The improved performance of the device prepared with Ni NWs (conduction band of ~3.87 eV), convincingly illustrated that Ni NWs TCE could effectively reduce the electron injection barrier from the anode to the electron injection layer, and thus increase the electron injection into the emitting layer. Moreover, we have also fabricated OLED utilizing Ag NWs as the bottom electrode. The operating voltage for Ag NWs is 3.4 V. The current, and power efficiencies are 13.28 cd/A, and 11.12 lm/W, respectively. The probably explanation for a higher operating due to the mismatch of the energy alignment between Ag NWs and PEDOT:PSS. In addition, our approach offers an effective strategy for preparing air-stable, efficient, and flexible display and lighting devices to replace the more brittle devices based on ITO electrodes. Based on our literature review, this is the first OLED realized with Ni NWs TCE.

Another possible demonstration has been realized by the fabrication of organic solar cells (OSCs). In this final demonstration, we employed poly[4,8-bis(5-(2-ethylhexyl)thiophen-2-yl)benzo[1,2-b;4,5-b′]dithiophene-2,6-diyl-alt-(4-(2-ethylhexyl)-3-fluorothieno[3,4-b]thiophene-)-2-carboxylate-2-6-diyl)] (PTB7-Th) and [6,^6^]-phenyl C_71_ butyric acid methyl ester (PC_71_BM) as our donor and acceptor, respectively. Our OSCs consist of Glass/Ni NWs/PEDOT:PSS/PTB7-Th:PC_71_BM/ZnO/Al.

**Figure 6:**
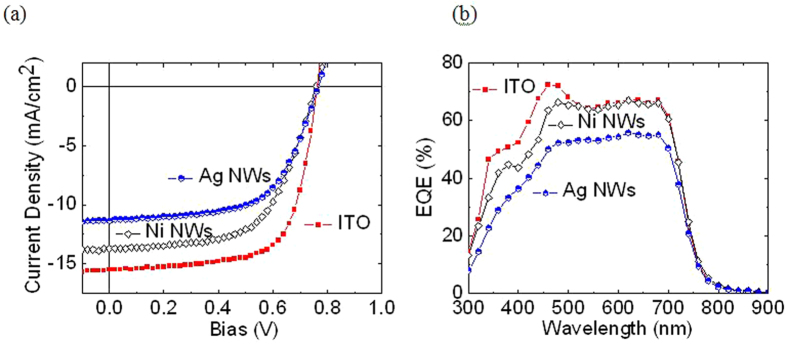
(**a**) J-V and (**b**) EQE characteristics of PTB7-Th:PC71BM based OSCs measured at 100 mW/cm2.

Figure 6 illustrates the typical current density-voltage and external quantum efficiency characteristics measured at 100 mW/cm^2^. Our proposed Ni NWs based OSC shows an efficiency of about 6.21% compared to the efficiency of ITO based OSC of 8.17% (Fig. 6a). Our proposed transparent electrode demonstrates relatively low J_sc_ and FF compared to the ITO transparent electrode due to low shunt and high series resistance. We believe that good morphology of the active layer is the key parameter for obtaining high FF. In general the series resistance indicates the integral conductivity of the device directly related to its internal carrier mobility while shunt resistance associated to the loss of current density via carrier recombination within the device, particularly at the interfaces of each layer. Shunt resistance is basically due to the manufacturing defects, rather than poor device design. High series resistance implies that the interphase contact is less desirable and the conductivity of every layer of the device is low. A low shunt resistance suggests that the power loss in the device through an alternate current path is very large, resulting in small FF. It is worth noting that the V_oc_ obtained in all devices are similar since it is solely determined by the HOMO and LUMO of the active materials used. The control devices fabricated employing Ag NWs show a slightly less performance to Ni NWs devices. The Ag NWs demonstrated a J_sc_ = 11.35 mA/cm^2^, a V_oc_ = 0.76 V, a FF = 60.70%, and a PCE = 5.24%. We attribute this undesirable device performance of Ag NWs to a higher RMS roughness of Ag NWs compared to that of Ni NWs (Ag NWs =  > 30 nm *vs*. Ni NWs =  <13 nm) and high R_S_ (possible causing short-circuiting issue) (See Supporting Information for more details of Ag NWs). Figure 6b shows the EQE of all devices, and we can see that the EQE of Ni NWs based OSC exceeded 60% in the visible. On the other hand, the EQE of Ag NWs is very much less compared to that of Ni NWs with an average of 50.98% in the visible region. Although, the performance of Ni NWs based OSC is slightly lower compared to that of the ITO based OSC, it is appealing for future application and could be used to replace our commonly used transparent electrode, ITO.

## Conclusions

In conclusion, we developed a novel approach to fabricating high performance transparent electrodes based on nickel nanowires. The nanowires are synthesized according to a facile direct template-free method. The sheet resistance of 19 Ω/sq. at 90% is obtained without any acidic treatment. The electrode fabrication process is carried out at room temperature from nanowires in solution. There is no need for post-treatments such as thermal annealing of forming gas. Moreover, we also demonstrate the fabrication of a highly efficient organic light emitting diode, comparing our proposed transparent electrode with a commercially available ITO electrode, in which we demonstrated that the performance of our proposed transparent electrode has outperformed the organic light emitting diode based on the ITO electrode.

## Additional Information

**How to cite this article**: Kim, J. *et al*. Organic devices based on nickel nanowires transparent electrode. *Sci. Rep*. **6**, 19813; doi: 10.1038/srep19813 (2016).

## Supplementary Material

Supplementary Information

## Figures and Tables

**Figure 1:**
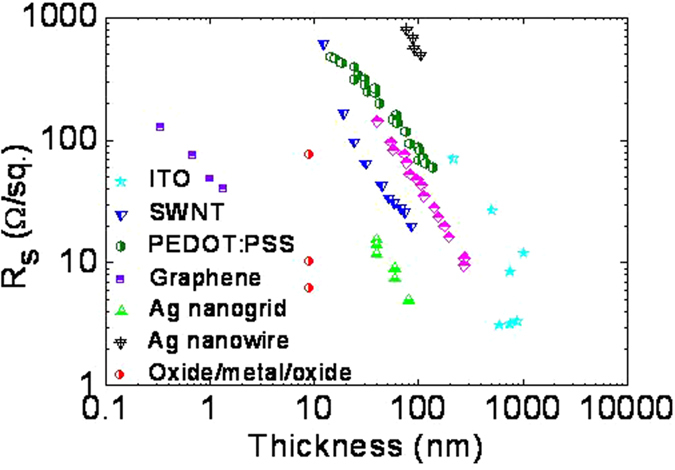
Sheet resistance (R_s_) as a function of film thickness for different TCE films: Ag, Al and Cu metal grids^30^; PEDOT:PSS^31^; ITO films^32^,^33^; SWNTs^32^; Ag nanogrid^32^; oxide/Ag/oxide films^34^; and graphene^32^.

## References

[b1] Kwak D.-J., Moon B.-H., Lee D.-K., Park C.-S. & Sung Y.-M. Comparison of Transparent Conductive Indium Tin Oxide, Titanium-Doped Indium Oxide, and Fluorine-Doped Tin Oxide Films for Dye-Sensitized Solar Cell Application. J. Electr. Eng. Technol. 6, 684–687 (2011).

[b2] Samad W. Z., Salleh M. M., Shafiee A. & Yarmo M. A. Transparent Conductive Electrode of Fluorine Doped Tin Oxide Prepared by Inkjet Printing Technique. Mater. Sci. Forum 663–665, 694–697 (2010).

[b3] Choi K.-H., Choi Y.-Y., Jeong J.-A., Kim H.-K. & Jeon S. Highly Transparent and Conductive Al-Doped ZnO/Ag/Al-Doped ZnO Multilayer Source/Drain Electrodes for Transparent Oxide Thin Film Transistors. Electrochem. Solid-State Lett. 14, H152–H155 (2011).

[b4] Zhou L., Chen X., Zhu F., Sun X. & Sun Z. Improving Temperature-Stable AZO–Ag–AZO Multilayer Transparent Electrodes using Thin Al Layer Modification. J. Phys. D: Appl. Phys. 45, 505103 (2012).

[b5] Roos A. Optical properties of pyrolytic tin oxide on aluminum. Thin Solid Films 203, 41–48 (1993).

[b6] Liu T., Kim D., Han H., Yusoff A. R. B. M. & Jang J. Fine-Tuning Optical and Electronic Properties of Graphene Oxide for Highly Efficient Perovskite Solar Cells. Nanoscale 7, 10708–10718 (2015). 26030146 10.1039/c5nr01433f

[b7] Yusoff A. R. B. M. . A high Efficiency Solution Processed Polymer Inverted Triple-Junction Solar Cell Exhibiting a Power Conversion Efficiency of 11.83%. Energy Environ. Sci. 8, 303–316 (2015).

[b8] Kim P.-Y., Lee J.-Y., Lee H.-Y., Lee S.-J. & Cho N.-I. Structure and Properties of IZO Transparent Conducting Thin Films Deposited by PLD Method. J. Korean Phys. Soc. 53, 207–211 (2008).

[b9] Kim H.-K. & Lim J.-W. Flexible IZO/Ag/IZO/Ag Multilayer Electrode Grown on a Polyethylene Terephthalate Substrate using Roll-To-Roll Sputtering. Nanoscale Res. Lett. 7, 67 (2012). 22222144 10.1186/1556-276X-7-67 PMC3275542

[b10] da Silva W. J., Yusoff A. R. B. M. & Jang J. GO:PEDOT:PSS for High-Performance Green Phosphorescent Organic Light-Emitting Diode. IEEE Electron Dev. Lett. 34, 1566–1568 (2013).

[b11] Kim H. M., Yusoff A. R. B. M., Youn J. H. & Jang J. Inverted Quantum-Dot Light Emitting Diodes with Cesium Carbonate doped Aluminium-Zinc-Oxide as the Cathode Buffer Layer for High Brightness. J. Mater. Chem. C 1, 3924–3930 (2013).

[b12] Shao Y., Xiao X., Wang L., Liu Y. & Zhang S. Anodized ITO Thin-Film Transistors. Adv. Funct. Mater. 24, 4170–4175 (2014).

[b13] Guo D. . Detecting Solution pH Changes Using Poly (N-Isopropylacrylamide)-co-Acrylic Acid Microgel-Based Etalon Modified Quartz Crystal Microbalances. Anal. Chim. Acta 773, 83–88 (2013). 22819053 10.1016/j.aca.2012.06.025

[b14] Hsu C.-S. . Ammonia Gas Sensing Performance of an Indium Tin Oxide (ITO) Based Device with an Underlying Au-Nanodot Layer. J. Electrochem. Soc. 160, B17–B22 (2013).

[b15] Patel N. G., Patel P. D. & Vaishnav V. S. Indium Tin Oxide (ITO) Thin Film Gas Sensor for Detection of Methanol at Room Temperature. Sen. Actuator B 96, 180–189 (2003).

[b16] http://www.technovio.com/pressrelease/increase-in-the-trade-of-indium-and-into-from-china-propelling-growth-in-the-global-ito. Increase in the trade of indium and ITO from china propelling growth in the global ITO market: Technavio Report. (Year published:2015) (Date access: 03/12/2015).

[b17] Kitaura R. . High-Yield Synthesis of Ultrathin Metal Nanowires in Carbon Nanotubes. Angew. Chem. 48, 8298–8302 (2009). 19774577 10.1002/anie.200902615

[b18] Yusoff A. R. B. M., Lee S. J., Schneider F. K., da Silva W. J. & Jang J. High-Performance Semitransparent Tandem Solar Cell of 8.02% Conversion Efficiency with Solution-Processed Graphene Mesh and Laminated Ag Nanowire Top Electrodes. Adv. Energy Mater. 4, 1301989–1301998 (2014).

[b19] Yusoff A. R. B. M., Kim D., Schneider F. K., da Silva W. J. & Jang J. Au-doped Single Layer Graphene Nanoribbons for a Record-High Efficiency ITO-Free Tandem Polymer Solar Cell. Energy & Environ. Sci. 8, 1523–1537 (2015).

[b20] Ellmer K. Past Achievements and Future Challenges in the Development of Optically Transparent Electrodes. Nat. Photonics 6, 809–817 (2012).

[b21] Langley D. . Flexible Transparent Conductive Materials based on Silver Nanowire Networks:A Review. Nanotechnology 24, 452001 (2013). 24121527 10.1088/0957-4484/24/45/452001

[b22] Krishnadas K. R., Sajanal P. R. & Pradeep T. Pristine and Hybrid Nickel Nanowires: Template- Magnetic Field-, and Surfactant-Free Wet Chemical Synthesis and Raman Studies. J. Phys. Chem. C 115, 4483–4490 (2011).

[b23] Kong Y. Y., Pang S. C. & Chin S. F. Facile Synthesis of Nickel Nanowires with Controllable Morphology. Mater. Lett. 142, 1–3 (2015).

[b24] Fu X. Y., Wang Y., Wu N. Z., Gui L. L. & Tang Y. Q. Preparation of Colloidal Solutions of Thin Platinum Nanowires. J. Mater. Chem. 13, 1192–1195 (2003).

[b25] Chen J. Y., Herricks T., Geissler M. & Xia Y. N. Single-Crystal Nanowires of Platinum can be Synthesized by Controlling the Reaction Rate of a Polyol Process. J. Am. Chem. Soc. 126, 10854–10855 (2004). 15339165 10.1021/ja0468224

[b26] Chen J. Y., Xiong Y. J., Yin Y. D. & Xia Y. N. Pt Nanoparticles Surfactant-Directed Assembled into Colloidal Spheres and used as Substrates in Forming Pt Nanorods and Nanowires. Small 2, 1340–1343 (2006). 17192984 10.1002/smll.200600015

[b27] Tiano A. L., Koenigsmann C., Santulli A. C. & Wong S. S. Solution-based Synthetic Strategies for One-Dimensional Metal-Containing Nanostructures. Chem. Commun. 46, 8093–8130 (2010). 10.1039/c0cc01735c 20848017

[b28] Koenigsmann C., Zhou W. P., Adzic R. R., Sutter E. & Wong S. S. Size-Dependent Enhancement of Electrocatalytic Performance in Relatively Defect-Free, Processed Ultrathin Platinum Nanowires. Nano Lett. 10, 2806–2811 (2010). 20608712 10.1021/nl100718k

[b29] Guo S. J., Zhang S., Sun X. L. & Sun S. H. Synthesis of Ultrathin FePtPd Nanowires and Their Use as Catalysts for Methanol Oxidation Reaction. J. Am. Chem. Soc. 133, 15354–15357 (2011). 21894999 10.1021/ja207308b

[b30] Lu X., Yavuz M. S., Tuan H.-Y., Korgel B. A. & Xia B. A. Ultrathin Gold Nanowires can be obtained by Reducing Polymeric Strands of Oleylamine−AuCl Complexes Formed via Aurophilic Interaction. J. Am. Chem. Soc. 130, 8900–8901 (2008). 18540574 10.1021/ja803343m

[b31] Huo Z., Tsung C.-K., Huang W., Zhang X. & Yang P. Sub-Two Nanometer Single Crystal Au Nanowires. Nano Lett. 8, 2041–2044 (2008). 18537294 10.1021/nl8013549

[b32] Kang M.-G., Guo L.J., Nanoimprinted Semitransparent Metal Electrodes and Their Application in Organic Light-Emitting Diodes. Adv. Mater. 19, 1391–1396 (2007).

[b33] Kim Y. H. . Highly Conductive PEDOT:PSS Electrode with Optimized Solvent and Thermal Post-Treatment for ITO-Free Organic Solar Cells. Adv. Funct. Mater. 21, 1076–1081 (2011).

[b34] Bonaccorso F., Sun Z., Hasan T. & Ferrari A. C. Graphene Photonics and Optoelectronics. Nat. Photon. 4, 611–622 (2010).

[b35] Fraser D. B. & Cook H. D. Highly Conductive, Transparent Films of Sputtered In_2−x_Sn_x_O_3−y_. J. Electrochem. Soc. 119, 1368–1374 (1972).

[b36] Holland L. & Siddall G. The Properties of Some Reactively Sputtered Metal Oxide Films. Vacuum 3, 375–391 (1953).

[b37] Mora S. B. S. & Cloutier S. G. Figures of Merit for High-Performance Transparent Electrodes Using Dip-Coated Silver Nanowire Networks. J. Nanomater. 2012, 286104–286110 (2012).

[b38] Mehra S., Christoforo M. G., Peumans P. & Salleo A. Solution Processed Zinc Oxide Nanopyramid/Silver Nanowire Transparent Network Films with Highly Tunable Light Scattering Properties. Nanoscale 5, 4400–4403 (2013). 23575765 10.1039/c3nr00863k

[b39] Preston C., Xu Y., Han X., Munday J. N. & Hu L. Optical Haze of Transparent and Conductive Silver Nanowire Films. Nano. Res. 6, 461–468 (2013).

[b40] Kumar A. B. V. K., Bae C. W., Piao L. & Kim S. -H. High Conductivity, Transparency, Adhesion and Low Haze. Mater. Res. Bull. 48, 2944–2949 (2013).

