# Supplementary material for: Organic devices based on nickel nanowires transparent electrode
Source: Sci Rep. 2016 Jan 25;6:19813. doi: 10.1038/srep19813 (PMC4726194; doi:10.1038/srep19813)
Supplement: Supplementary Information [file srep19813-s1.doc]

**Supporting Information**

Organic devices based on nickel nanowires transparent electrode

Jeongmo Kim,^a^ Wilson Jose da Silva,^b^ Abd. Rashid bin Mohd Yusoff,^a^ and Jin Jang*^a^

^a^Advanced Display Research Center, Department of Information Display, Kyung Hee University, Dongdaemoon-gu, Seoul 130-701, South Korea. Email: jjang@khu.ac.kr

^b^Universidade Tecnologica Federal do Parana, GPGEI – Av. Sete de Setembro, 3165 – CEP 80230-901 – Curitiba, Parana, Brasil: Email: wjsilva2000@yahoo.com.br

**Experimental section**

*Nickel nanowires solution preparation*

All chemical reagents in our experiments are analytical grade and are used without further purification. Nickel (II) chloride hexahydrate (NiCl_2_•6H_2_O) with 0.08 mol/L was dissolved in a 100 mL mixture solution of distilled water and ethanol with a volume ratio of 5:4. 0.2 mL hydrazine hydrate (NH_2_NH_2_•H_2_O), as a reducing agent with concentration of 85 wt%, was added into the mixture during vigorous stirring. Later, 5 M sodium hydroxide (NaOH) solution was deployed to adjust the pH value to 13. The as-prepared, blue transparent solution was kept at 50 °C for 20 min. A permanent NdFeB magnet was place under the flask. Throughout this work, we used a magnetic field of 0.5 T for 30 min. A permanent magnet was placed with different applied magnetic fields and the magnetic field was measured using a Gauss meter (THM1176 Three-axis hall magnetometer). Different magnetic fields were obtained by controlling the distance between the flask and the magnet. Finally, the grey-black fluffy solid product floated to top of the solution surface and the solution became colorless and transparent. The product was collected and washed several times using distilled water and ethanol under an applied magnetic field and then dried in a vacuum oven at 60 °C for 12 h.

*Silver nanowires*

Ag NWs was obtained from Blue Nano Inc. with an average diameter of 90 nm and length of 10-30 μm. The obtained concentration was 10 mg/mL which was diluted to desired concentrations prior casting as films.

*Characterizations*

Powder X-ray diffraction (XRD) patterns were recorded using a Bruker diffractometer with Cu Kα radiation (D8 Advance X-ray diffractometer, Cu Kα, λ = 1.5406 Å, 40 kV, and 40 mA) to study the crystallographic information of the samples. Field-emission scanning electron microscopy (FESEM; JEOL, JSM-6700F, 5 kV) equipped with energy-dispersive X-ray spectroscopy (EDX) was used to analyze the morphology and elemental composition of the samples. The surface properties of the samples were analyzed with an X-ray photoelectron spectroscopy (XPS; VG ESCALAB MKII instrument) that uses a Mg Kα X-ray source. The survey scans were collected using a pass energy of 160 eV. High-resolution spectra of the individual elements were collected with the analyzer pass energy set at 40 eV. The pressure of the analyzer chamber was maintained at 10^−9^ Pa during the measurement. Before the analysis, all the samples were dried under a vacuum at 80 °C overnight. Raman scattering experiments were performed at room temperature using a Ramanor T-64000 microscopy system (Jobin Yvon, Longjumean, France). Contact angle measurement was performed using an Attension Theta optical tensiometer with automated liquid pumping system was used for the contact angle measurements. Purified (Milli-Q) and degassed water was used as the probe liquid.

*Fabrication of OLED*

OLEDs were fabricated on Ni NWs-coated glass substrates with a sheet resistance of 19 Ω/sq. Before device fabrication, the glass substrates were cleaned with Decon 90, rinsed in de-ionized water, dried in an oven at 120°C, treated with UV-ozone, and transferred to a vacuum deposition system with a base pressure better than 1×10^−6^ mbar for organic and metal deposition. The devices were fabricated by evaporating organic material onto the PEDOT layer sequentially with an evaporation rate of 1–2 Å/s .The cathode was completed through a thermal deposition of LiF at a deposition rate of 0.1 Å/s, and then capped with an Al metal through thermal evaporation at a rate of 10 Å/s. The current−voltage−luminance characteristics were measured using a Keithley 236 source measurement unit and a Minolta CS2000 Spectroradiometer.

*Fabrication of OSC*

OSCs were developed on Ni NWs-coated glass substrate with a sheet resistance of 19 Ω/sq. Before device fabrication, the glass substrates were cleaned with Decon 90 and rinsed in de-ionized water. Then a 30 nm thick PEDOT:PSS (Argos Organic Chemicals) hole conducting layer was spin-casted onto the Ni NWs electrode (4000 rpm, 1 min). The samples were then baked at 115 °C for 15 min on a hot plate. A bulk heterojunction (BHJ) layer was spin-casted onto the PEDOT:PSS layer. The solution was prepared by dissolving poly({4,8-bis[(2-ethylhexyl)oxy]benzo [1,2-b:4,5-b’]dithiophene-2,6-diyl}{3-fluoro-2-[(2-ethylhexyl)carbonyl]thieno[3,4-b]thiophenediyl}) (PTB7-Th) and [6,6]-phenyl C_71_ butyric acid methyl ester (PC_71_BM) in a 1:1.5 weight ratio in a 3% diiodooctane-containing chlorobenzene solvent. A 6 nm thick ZnO layer was coated onto the BHJ layer. Finally, a 100 nm thick Al cathode was thermally evaporated under 10^−7^ Torr. The J−V performances of the OSCs were measured using an Oriel 91193 (1 kW lamp, 100 mW/cm^2^) device, an NREL-calibrated Si solar cell, and Keithley 2400 source meters. An aperture was used to determine the cell area. The incident photon-to-current efficiency (IPCE) measurements were conducted using a Solar Cell QE/IPCE (Zolix Solar Cell Scan 100).

(a)

(b)

**Figure S1.** Diameter (a) and length (b) distributions determined from 72 Ni NWs. A relatively high number in (b) * probably comes from nanowire breaks during the purification step.

**Figure S2.** Transmittance *vs.* Ni NWs network density.


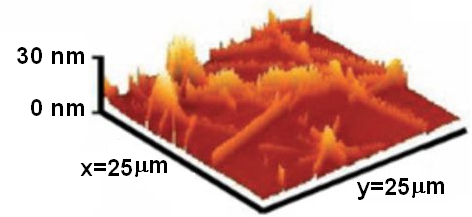

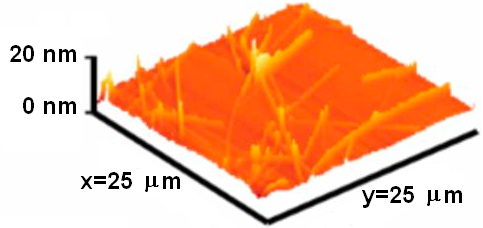


**Figure S3.** AFM images of the Ag NWs (upper panel) and Ni NWs (down panel).
